# The Role of Incubation Conditions on the Regulation of Muscle Development and Meat Quality in Poultry

**DOI:** 10.3389/fphys.2022.883134

**Published:** 2022-06-15

**Authors:** Yuan-Hao Wang, Jing Lin, Jing Wang, Shu-Geng Wu, Kai Qiu, Hai-Jun Zhang, Guang-Hai Qi

**Affiliations:** Laboratory of Quality and Safety Risk Assessment for Animal Products on Feed Hazards (Beijing) of the Ministry of Agriculture and Rural Affairs, Research Institute of Feed, Chinese Academy of Agricultural Sciences, Beijing, China

**Keywords:** poultry, incubation condition, myofiber formation, muscle development, meat quality

## Abstract

Muscle is the most abundant edible tissue in table poultry, which serves as an important source of high protein for humans. Poultry myofiber originates in the early embryogenic stage, and the overall muscle fiber number is almost determined before hatching. Muscle development in the embryonic stage is critical to the posthatch muscle growth and final meat yield and quality. Incubation conditions including temperature, humidity, oxygen density, ventilation and lighting may substantially affect the number, shape and structure of the muscle fiber, which may produce long-lasting effect on the postnatal muscle growth and meat quality. Suboptimal incubation conditions can induce the onset of myopathies. Early exposure to suitable hatching conditions may modify the muscle histomorphology posthatch and the final muscle mass of the birds by regulating embryonic hormone levels and benefit the muscle cell activity. The elucidation of the muscle development at the embryonic stage would facilitate the modulation of poultry muscle quantity and meat quality. This review starts from the physical and biochemical characteristics of poultry myofiber formation, and brings together recent advances of incubation conditions on satellite cell migration, fiber development and transformation, and subsequent muscle myopathies and other meat quality defects. The underlying molecular and cellular mechanisms for the induced muscle growth and meat quality traits are also discussed. The future studies on the effects of external incubation conditions on the regulation of muscle cell proliferation and meat quality are suggested. This review may broaden our knowledge on the regulation of incubation conditions on poultry muscle development, and provide more informative decisions for hatchery in the selection of hatching parameter for pursuit of more large muscle size and superior meat quality.

## 1 Introduction

Poultry is major source of protein and the demand of poultry meat and other by-products are increasing day-by-day throughout the world (Scanes, 2007). With the rapid development of modern society, consumers are demanding more and more quality poultry meat. In the past decades, great progress in genetic selection and management has been made in meat type poultry, which contribute to the fast growth rates and large muscle mass (Petracci et al., 2015). However, the increasing production of poultry meat has been accompanied by increasingly serious meat quality problems including white striping, woody breast and spaghetti meat (Baldi et al., 2018; Oviedo-Rondón et al., 2020; Che et al., 2022), which will negatively affect the desire of people to consume. As a result, there is growing interest in searching for new approaches to improve poultry meat quality while increasing the meat yield.

Incubation phase is a critical stage in the life cycle of poultry, which covers the phase from the start of embryogenesis to the beginning of the young bird stage or birth (Gilbert and Knisely, 2000) and play an important role in the skeletal muscle growth and final meat quality in poultry (Nyuiadzi et al., 2020; Guo et al., 2021). Skeletal muscle is the dominant component of poultry meat. It is well established that poultry muscle fiber number is determined during embryonic development (Stockdale and Miller, 1987). Evidences have shown that regulation of myofiber development during myogenesis can increase the number and fiber diameter of muscle fiber (Gonzalez and Jackson, 2020; Ma et al., 2020). Therefore, environmental regulation during the incubation phase may be a novel way out to ameliorate the muscle development and meat quality in poultry.

Incubation conditions including temperature, humidity, lighting, ventilation and other regimen affects the development of muscle. The effect of changing incubation conditions on embryonic development and muscle growth in birds has gained much attention in the fields of poultry science in recent years. It was demonstrated that lighting and thermal adjustment during the incubation phases improves the number and activity of satellite cells, promotes muscle fiber development, increases the embryonic muscle fiber area (Zhang et al., 2014) and improved meat quality (Piestun et al., 2013). Incubation of chicken embryos in a hypoxic environment with 17% oxygen content improves posthatch broiler body weight and increases pectoral muscle production (Druyan et al., 2018). Green light exposure during broiler incubation increased the number and proliferative activity of myogenic and satellite cells, promoted embryonic muscle fiber development (Bai et al., 2019), and increased pectoral muscle production (Zhang et al., 2012). Heating at 39.5°C for 12 h per day from embryonic day E7 to E16 increased the weight and ratio of embryonic pectoral muscle and improved the number and proliferative activity of adult myoblasts (Piestun et al., 2013). Stimulation of embryonic eggs at 39.5°C for 3 or 6 h per day during the E16–E18 stages increased broiler pectoral muscle production myofiber diameter (Piestun et al., 2009). When the incubation temperature was adjusted upward to 38.8°C at mid-embryonic stage, the pH values and water holding capacity of the breast muscle after slaughter were improved (Janisch et al., 2015). Increasing the temperature to 39.5°C can reduce the severity of many microscopic features of myopathies common in the broiler industry and have a positive effect on improving muscle quality (Clark et al., 2017). If the incubation environment is suboptimal, it is not conducive to the formation of excellent chicken meat quality. Irradiation of the embryos at 39.2°C for 3 h per day at E8–E10 and E16–E18, respectively, decreased the water holding capacity and pH values of the broiler breast meat (Collin et al., 2007). It is clear that regulation of the incubation environment can be a potential solution for improving poultry muscle quality during poultry breeding.

Therefore, the purpose of this paper is to present scientific evidence that incubation conditions may convey substantial and lasting effects on embryonic muscle fiber and posthatch muscle growth as well as meat quality at market age. The first part will describe the muscle fiber growth in poultry, with an emphasis on the hormone and growth factor. The second section will provide scientific results of incubation parameter-induced changes of muscle growth and meat quality, and discuss the modes of action for diverse incubation conditions. The third portion will summarize the overall evidences and recommend future directions for incubation condition modulation on embryonic muscle fiber development and meat quality in poultry.

## 2 Physiological Processes of Muscle Fiber Development

### 2.1 Developmental Characteristics of Embryonic Muscle Fibers

The embryonic stage is an important period for the formation of muscle fibers. Embryonic muscle fibers are differentiated from myogenic precursor cells. Myogenic precursor cells proliferate and differentiate into myogenic fibers, move toward the site of muscle formation, align and fuse to form myotubes, and finally fuse into multinucleated myofibers, which in turn fuse with existing muscle fibers to form new myofibers, and further differentiate into skeletal muscle (Swartz et al., 1994). Myoblasts can be designated as embryonic, fetal and adult (Stockdale, 1992), embryonic myoblasts differentiate into primary fibers, fetal myoblasts form secondary fibers and adult myoblasts plays an important role in the formation and growth of muscle fibers during late incubation period and after hatching of poultry (Stockdale and Miller, 1987). On E14, a third type of myogenic cells (satellite cells) emerge, which attach to myofibrils beneath the basement membrane of muscle fibers (Mauro, 1961; Hartley et al., 1992). Satellite cells have the potential to promote postnatal skeletal muscle growth in vertebrates, and are the main source of myoblasts in neonatal skeletal muscle growth (Moss and Leblond, 1970). The number of satellite cells rapidly decreases 1 week after hatching. When muscle is stimulated, the activated satellite cells proliferate and differentiate to fuse with the original myofibers to form new muscle fibers (Hawke and Garry, 2001; Halevy et al., 2004; Halevy et al., 2006a; Allouh et al., 2008).

### 2.2 Effects of Hormones and Myogenic Regulatory Factors on Muscle Fiber Development

Hormones also have the potential to promote the development and growth of skeletal muscle during embryonic development. In addition, hormones can also influence embryonic development through multiple mechanisms, such as muscle cell growth, proliferation, and differentiation (Dishon et al., 2017, 2018). It has been found that hypertrophy of muscle fibers during late hatching may be associated with increased levels of thiothyrotropine (T3) and thyroxine (T4), thyroid hormones. Among them, T3 and T4 are involved in many physiological processes, including embryonic muscle growth (Christensen et al., 1996) and stimulation of embryonic hatching (Christensen et al., 2003, 2004). Regulating specific environmental factors can ameliorate physiological response at the critical stages of embryonic development. Fertilized duck eggs placed in environment with 1% CO_2_ before incubation had a positive effect on posthatch body weight by increasing T3, T4 and corticosterone levels in plasma of embryos (El-Hanoun et al., 2019). In mammals and birds, the proliferation and differentiation of muscle cells are controlled by growth factors and other factors that play an important role in promoting skeletal muscle growth (Adams et al., 1999). The mechanism controlling muscle growth and development is the pro-growth axis, which includes hypothalamic growth hormone-releasing hormone (GHRH), growth hormone (GH) produced by the anterior pituitary, insulin-like growth factor 1 (IGF-1) produced by the liver and skeletal muscle, and the corresponding receptors. GH and IGF-1 induce proliferation and differentiation of muscle cells (Halevy et al., 2006b). In later incubation period, GH levels are high in blood and decline with age (Buyse and Decuypere, 1999; Kim, 2010). GH affects growth by activating its receptor (Edens and Talamantes., 1998). The growth hormone receptor (GHR) is a transmembrane protein that begins to develop on E12 (Kocamis et al., 1999), and can be found in a variety of tissues, including liver, muscle, and adipose tissue (Mao et al., 1998). Receptors in muscle can be found on satellite cells, and irradiating embryonic eggs with green light during incubation increases GHR expression on satellite cells (Halevy et al., 2006b). IGF-1 is one of the most important growth factors regulating satellite cell proliferation, and it plays a role in muscle growth and hypertrophy through its effects on satellite cells (Adams and Mccue., 1998; Adams et al., 1999). IGF-1 has also been found in amniotic fluid and may play a role in embryonic regulation of amino acid utilization (Karcher et al., 2005). IGF-1 belongs to a family of insulin-related peptide hormones with multiple metabolic and anabolic properties. IGF-1 play an important role in the metabolism of carbohydrates, lipids and proteins in a variety of tissues, including liver, muscle and adipose tissue (Kanacki et al., 2012), and is able to stimulate hepatic glycogen, RNA and protein synthesis (Mcmurtry, 1998). In addition, it also plays a key role in muscle cell proliferation and differentiation. Similar to GHR in satellite cells, muscle IGF-1 was also significantly elevated in birds exposed to light stimulation in the early post-hatching period (Halevy et al., 2006b). The expression level of *IGF-1* gene in broiler pectoral muscle was higher when the incubation temperature is increased to 39.5°C at E12 to E18 embryonic ages (Piestun et al., 2009).

The proliferation and differentiation process of myogenic cells is controlled by a family of muscle-specific basic helix-loop helix transcription factors containing four basic helix-loop-helix *myogenic differentiation antigen* (*MyoD*), *myogenic factor-5* (*Myf5*), *myogenin* (*MyoG*) and *myogenic regulatory factor-4* (*MRF4*) that positively regulate myogenesis. These factors are sequentially expressed when satellite cells are activated (Weintraub, 1993) and are essential for satellite cell activation, proliferation and differentiation (Schultz and Mccormick., 1994). *MyoD* and *MyoG* are only continuously expressed in activated satellite cells (Hernández-Hernández et al., 2017). Numerous studies have shown that *MyoD* is a marker of satellite cell activation and proliferation, while *MyoG* is a marker of cells entering the terminal differentiation program (Smith et al., 1994; Seale and Rudnicki, 2000). Higher myogenic inhibitor mRNA levels are accompanied by lower *MyoD* and *MyoG* mRNA levels, and myogenic inhibitor inhibits muscle growth by downregulating the gene expression of *MyoD*, *Myf5*, and *MyoG* (Thomas et al., 2000; Langley et al., 2002). Initially, *Myf5* and *MyoD* are expressed in proliferating zygotes, and then *MyoG* is also expressed as cells begin to differentiate (Beauchamp et al., 2000). Increasing the incubation temperature (39.5°C, 3 and 6 h) between E12 and E18 embryonic has been reported to promote cell differentiation and increase muscle myostatin expression levels (Piestun et al., 2009). Hypoxia during embryonic development inhibits myogenic cell differentiation, decreases the expression of *MyoD*, *Myf5* and myosin heavy chain, and hinders the formation of multinucleated myotubes (Beaudry et al., 2016; Yang et al., 2017). *Paired box 7* (*Pax7*) is considered an early marker of myogenesis during post-hatching muscle growth, and is selectively expressed in quiescent and proliferating satellite cells and is essential for their self-renewal (Halevy et al., 2004; Allouh et al., 2008). Furthermore, the expression of *Pax7* was increased during myogenic cell proliferation and decreased during differentiation (Halevy et al., 2004). Previous studies have reported that increasing the incubation temperature to 39.5°C during E12–E18 increased the proliferative activity of adult myoblasts, thymidine DNA content, the number of muscle cells with PCNA in the muscle, and *Pax7* protein expression level (Piestun et al., 2009).

### 2.3 The Effect of Muscle Fiber Development on Meat Quality

The growth and development rate of muscle tissue before and after hatching has an important influence on muscle yield and meat quality after hatching and even at market age (Gratta et al., 2019; Ma et al., 2020). The number and morphology of different fibers are closely related to meat quality, when the number of small diameter fibers in the muscle is high and the intramuscular fat is abundant, the meat more tender. Conversely, when the muscle has a higher proportion of fibers with larger cross-sectional area and increased glycogen content, the pH values of muscle decreases rapidly after slaughter, the final pH values and tethering power are lower, and the color and quality of the meat may be slightly worse (Joo et al., 2013; Cong et al., 2017). During mid-incubation, increasing the incubation temperature to 38.8°C increased post-slaughter pectoral muscle pH values and tethering power and improved final meat quality in broilers (Janisch et al., 2015). Poultry meat quality can be affected by pectoral muscle diseases, including pectoral woody meat, white striping and spaghetti meat, which are manifested in poor muscle microvascularization, leading to muscle fiber degeneration during muscle regeneration, with a rounded shape and internalized nuclei (Soglia et al., 2016a; Sihvo et al., 2018; Petracci et al., 2019). These lesions can be observed in broilers during the first week after hatching (Chen et al., 2019), with diffuse thickening of the endomysium and perimysium connective tissue of granulation tissue, increased deposition of connective tissue (fibrosis), increased fat deposition or infiltration, leading to varying degrees of muscle damage in the pectoral muscle. (Soglia et al., 2016b; Clark and Velleman, 2016). Among them, woody meat showed the appearance of sclerotic and uniform pale areas in the muscle, white striping meat showed the presence of white streaks parallel to the direction of the muscle fibers, and spaghetti meat showed the tendency to separate the fiber bundles that make up the muscle tissue of the breast muscle (Soglia et al., 2019). Increasing the incubation temperature from 37.8 to 39.5°C for 12 h a day during the late incubation period can reduce the severity of many microscopic features of myopathies common in the broiler industry (Clark et al., 2017), which may have a positive effect on improving meat quality and processing functions.

### 2.4 Environmental Regulation Effect on Muscle Fiber and Meat Quality

#### 2.4.1 Temperature on Muscle Development

Temperature is the most important environmental factor to regulate the embryonic development (Fasenko, 2007). Variations in incubation temperature in stages of embryonic muscle fiber development have diverse effects on muscle fiber development and posthatch muscle growth. Generally, mild increase of temperature can stimulate muscle fiber growth, while severe high temperature tends to cause embryo death (Reyna and Burggren, 2012). Piestun et al. (2015) demonstrated that temperature elevation during mid-term embryogenesis results in enhanced muscle growth in the embryo and posthatch by induction of myoblasts proliferation. Temperature stimulation aims to improve embryonic muscle fiber and posthatch muscle development especially in mid-incubation period, when the myogenic cells proliferate rapidly, which can determine the final muscle fiber number, morphology, and size (Stockdale, 1992). Broiler muscle yield was reported to be improved when E7-E16 embryos were indirectly incubated at high temperature (39.5°C) for 12 h per day, due to the elevation of number and proliferative activity of pectoral myoblast. (Piestun et al., 2013). From E7 to E16, the 24 h continuous high temperature exposure at 39.5°C reduced broiler body weight, but it increased breast muscle percentage and yield (Piestun et al., 2015). This may be related to the difficulty of dissipating excess heat in the embryo, which leads to teratogenic consequences and growth retardation regardless of the increased proliferation of myoblasts, compared to the sustained effect of intermittent high-temperature stimulation on myofiber development at mid-hatch without affecting the final body weight of the broiler, and increased pectoral muscle production. Krischek et al. (2018) found that increased the incubation temperature from 37.8 to 38.8°C from E7 to E16 increased the muscle fiber area at hatching and body weight of broiler at 35 D, indicating that the positive effect of increasing the temperature at mid-incubation on embryonic muscle fiber development could persist until market age.

Similarly, exposed embryonic eggs to 38.5 and 39.5°C conditions for 18 h per day between E12 and E18 was observed to increase the posthatch broiler body weight and pectoral muscle production by upregulating muscle growth factor (IGF-1 and GH) and muscle marker gene (*MyoD*, *MyoG*, *Pax7*, and *PCNA*) expression during incubation and posthatch (Al-Zghoul and El-Bahr, 2019). Late hatching is a critical period for the development of satellite cells, the only myogenic cells capable of repairing damaged muscle fibers and promoting post-muscle growth, and they are able to re-enter the cell cycle under different muscle stresses, proliferate, and then fuse into existing or newly formed muscle fibers (Hawke and Garry, 2001). At E16–E18, stimulating embryonic eggs at 39.5°C for 3 or 6 h per day increased the number and activity of embryonic and number of myoblasts in broilers before and after hatching, and pectoral muscle fiber diameter on 13 day of age (D) and 35D, as well as the final body weight and pectoral muscle production in broilers (Piestun et al., 2009). Thus, it is clear that increasing the incubation temperature appropriately during the critical stage of muscle fiber development has a direct and lasting positive effect on embryonic muscle fiber development and post-hatch muscle growth. In another study, heating (38.1°C) the embryos between embryonic E0 and E5 of incubation, increased the body weight of broiler chicks on the day of hatching and at 35D, and significantly increased pectoral muscle production and plasma testosterone levels (Lin et al., 2017). As an important sex hormone, testosterone levels are positively correlated with broiler growth, and high plasma testosterone levels are associated with faster growth and greater body weight (Buyse et al., 1996), whether raising high incubation temperatures in the pre-hatching period is associated with the ability to promote muscle development by increasing plasma testosterone levels needs further study.

Studies regarding the effect of incubation temperature on meat quality are fewer and results vary. Pre-hatching thermal stimulation at 39.5°C for 3 h per day reduced breast muscle pH values, while continuous stimulation between E 8–10 and E16–E18 reduced pH values and water holding capacity of chicken meat after slaughter (Collin et al., 2007). Temperature adjustment upward to 38.8°C in the middle stage of incubation, improved the broiler breast meat quality as evidenced by the increased post-slaughter breast muscle pH values and reduced grill loss (Janisch et al., 2015). Increasing the incubation temperature from 37.8 to 39.5°C for 12 h per day during late incubation reduced the severity of many microscopic features of myopathy common in the broiler industry (Clark et al., 2017), and myopathy severity and muscle degradation had a significant negative effect on meat quality and processing characteristics (Soglia et al., 2016a). Therefore, increasing the incubation temperature to below 39.5°C for intermittent thermal manipulation during late embryonic development can be a viable management strategy to improve meat quality and processing functions.

#### 2.4.2 Lighting on Muscle Development

Light is an important exogenous environmental factor that affects embryonic development during incubation. As early as the late 1960s, it was found that embryonic development was accelerated when fertilized eggs were stimulated by continuous light (Siegel et al., 1969). Studies have shown that providing light in a dark incubation environment has a positive effect on embryo development, while different colors of light during incubation has diverse effects on embryo development and muscle growth after emergence (Rozenboim et al., 2003, 2004).

Green light stimulation during incubation is known to be a method to increase poultry meat production. Green light illumination to embryos during incubation increases turkey body weight at 28 D, and this positive effect can be sustained up to 79 D, increasing breast muscle production (Rozenboim et al., 2003). Green light stimulation given during the incubation period had strongest effect on muscle growth (Rozenboim et al., 2004). Green light during incubation of chicken embryos increased daily weight gain and pectoral muscle weight in the week before fledging, which was beneficial for early development and muscle growth of broiler chicks. (Özkan et al., 2012a). Green light stimulation of chicken embryos during incubation increased pectoral muscle and embryo weight during incubation, and also increased the proportion of pectoral muscle during early chick emergence (Rozenboim et al., 2004).

Monochromatic green light irradiation at different incubation periods increased GH levels in plasma and up-regulated hypothalamic growth-promoting axis gene expression of chicken embryos (Dishon et al., 2021), Green light stimulation at E0 increased GH levels in the plasma of chicken embryos from E14 to E20, and upregulated GHR and insulin growth factor I gene expression in the liver, GH can act through GHR in muscle and adipose tissues, and through pro-growth axis mechanisms to influence embryonic development, such as muscle cell growth, proliferation and differentiation (Dishon et al., 2017). Intermittent light irradiation from E5 to E14, followed by continuous green light stimulation, up-regulated Pax7 and myostatin expression, increased the number of pectoral myogenic cells and the proliferation activity of satellite cells, promoted the proliferation and differentiation of myogenic cells, and increased the posthatch breast muscle weight of broiler chicks (Halevy et al., 2004). In addition, green light stimulation in incubation phase could up-regulate the gene expression of *MyoG* and *MyoD* in chicks, thus leading to the increase of cross-area of muscle fiber and weight of breast muscle 1 week after hatching (Zhang et al., 2012, 2014). Therefore, muscle growth-promoting effect of green light irradiation may be related to the regulation of growth-promoting hormone secretion and muscle growth factor gene expression.

In addition to green light, other colors of light during the embryonic period also positively affect chicken embryo development and posthatch muscle growth. White light stimulation during E14–E21 increased the proportion of breast muscle at hatching (Özkan et al., 2012b). Blue light stimulation during the incubation period had no effect on posthatch body weight and muscle mass of broiler chicks (Zhang et al., 2012), but another study showed that blue light stimulation during the embryonic period increased the average posthatch body weight of broiler chicks (Li et al., 2021), and the reason for this occurrence may be related to factors such as the type and size of embryonic eggs and the age of the breeder hens. Overall, it is demonstrated that light stimulation can promote muscle fiber development and muscle growth, but it may cause changes in muscle composition and impair muscle quality when broiler growth rate is too fast (Duclos et al., 2007). Continuous green light stimulation during incubation did not have any negative effect on muscle composition and meat quality of broilers at market age (Zhang et al., 2012).

#### 2.4.3 Oxygen and Carbon Dioxide on Muscle Development

Generally, the concentration of oxygen in the incubator is maintained at 21% (De Smit et al., 2006), but the concentration of carbon dioxide in the incubator should be maintained within the range of approximately 0.1–0.5% (Buys et al., 1998). The process of hypoxia is a normal part of fetal life in all vertebrates, when the embryo is exposed to hypoxic stress, blood vessels in the tissues begin to grow and promote vascular development to meet the demand for oxygen to the embryo (Druyan and Levi, 2012). Mild hypoxia leads to a decrease in oxygen consumption in chicken embryos and affects hatching weight. Prolonged hypoxia can affect embryonic viability (Szdzuy et al., 2008) and can self-regulate through functions such as inhibition of tissue growth, and this adverse effect may manifest itself at later stages of hatching or growth, affecting embryonic growth and development, especially at the beginning and end of hatching (Decuypere et al., 2002; Lundy, 1969).

Subjecting chicken embryos to low oxygen conditions with 14% oxygen content from E0 to E10 reduced embryonic and hatching body weights (Miller et al., 2002). Incubation of embryonic eggs at high altitude conditions reduced broiler body weight on 14D and inhibited pre-growth, but did not affect final body weight (Hassanzadeh M, 2004). However, incubation of Dwarf chickens at an altitude of 2,900 m significantly reduced hatchability and shell weight. Increasing oxygen concentration to 27.5% during mid and late incubation phase improved this situation and increased broiler hatching weight (Zhang et al., 2008). In contrast, exposing embryos to a low oxygen (17%) environment at E5–E12 increased 7D broiler body weight, a positive effect that lasted until 28D, and increased breast muscle production (Druyan et al., 2018). This occurred probably because the low-oxygen environment stimulated the embryonic vasculature to develop in a good direction and to better deliver nutrients to the pectoral muscle, (Hadad et al., 2014), thus promoting posthatch muscle development.

Carbon dioxide is an important gas during embryo development and bird egg incubation, and developing embryos require different levels of carbon dioxide at specific developmental stages and is an essential factor affecting embryo development (Mortola, 2009). Placing well-developed embryos in a CO_2_ incubator at a concentration of 4% at E10–E18 reduced embryo weight, but had no effect on chick weight on the day of fledging or day-old chicks (Everaert et al., 2007). However, placing fertilized duck eggs in an incubator with 1% CO_2_ content 10 days before hatching increased T3, T4 and corticosterone levels and body weight in plasma of Pekin ducks at embryonic stage and on the day of hatching, with positive effects on body weight lasting until market age (El-Hanoun et al., 2019). Thyroid hormones play an important regulatory role in maintaining chicken embryo development and normal development and can promote embryonic development (King et al., 1984). It was reported that 1% concentration of CO_2_ may affect embryonic muscle fiber development and posthatch muscle growth by raising blood levels that may stimulate plasma corticosterone and T3, leading to the onset of hatching (De Smit et al., 2006), thereby increasing Pekin duck embryo and market-day weight.

#### 2.4.4 Humidity on Muscle Development

Water release from the embryo to the external environment in the form of water vapor due to the electrical conductivity of the eggshell surface and the presence of stomata is inevitable and necessary throughout the incubation phase (Christensen et al., 2005). However, excessive water loss from embryonic eggs during incubation affects embryo development (Rahn and Ar, 1974), and improving this problem by changing the incubator humidity can positively affect embryo development and increase chick weight (Peebles and Brake, 1987; Swann and Brake, 1990). The optimal incubation humidity range has been reported to be quite wide, between 40 and 70% humidity (Lundy, 1969). Incubation of embryonated eggs at 53% or 63% humidity increased the hatching weight of broiler chicks compared to the low humidity group (43% humidity) (Bruzual et al., 2000). During incubation, the optimal weight of Pekin ducks on the day of hatching and at 21D was achieved only at 60, 65 and 70% of incubation humidity with increasing parental age (El-Hanoun et al., 2012). The incubation humidity of 63% increased the crude protein and crude fat content in the embryos while increasing the weight of the embryos at the later stages of hatching (Peebles et al., 2001). The increase in crude protein content in chicken embryos may be related to the acceleration of embryonic muscle development induced by the increased humidity. Although an increase in embryonic humidity can increase crude protein and crude fat content in chicken embryos, resulting in changes in the nutritional composition of chicken embryos, it is unclear whether changes in embryonic humidity can affect the nutritional composition of muscle in broiler chickens at market age and thus improve meat quality.

### 2.5 Other Factors on Muscle Development

#### 2.5.1 Holding Time of Fertile Egg

Holding time of fertile egg before hatching is also an important factor to affect embryonic development (Pokhrel et al., 2018). Embryonic eggs can be maintained at a specific embryonic stage after leaving the mother, when the embryo temporarily stops significant metabolic activity or development (Fasenko, 2007), but embryonic eggs can only be preserved for a certain developmental period (Pokhrel et al., 2021). Pearl chick embryos stored for 5 days improved their hatching day and final body weight after hatching compared to the counterparts stored for 10 days (Kouame et al., 2021). Compared to 12 days of storage for embryonic egg, 5 days of storage increased the hatching weight of broilers, and this weight promotion effect lasted until 42D (Damaziak et al., 2018). Similarly, embryos stored for 4 days had higher body weights after hatching compared to embryos stored for 21 days (Dymond et al., 2013). Another study showed that the embryonic development of fertilized eggs stored for 14 d was found to be delayed by about 12.2 h compared to embryonic eggs that were not stored (Mather and Laughlin, 1977). Long-term storage of embryonic eggs can cause embryonic stress, resulting in embryonic necrosis and reduced cell numbers (Arora and Kosin., 1968; Bloom et al., 1998), which can cause serious damage to the embryo, as well as altering the albumin pH in the embryonic eggs (Karoui et al., 2006), affecting the recovery of the embryonic eggs and reducing the hatchability and chick quality (Hamidu et al., 2011; Akhlaghi et al., 2013). Therefore, with the increase of embryonic egg storage time, there is a developmental lag in the embryo during incubation, which makes the embryo development slower and thus affects the muscle fiber development, which is not conducive to muscle growth after hatching.

#### 2.5.2 Short Periods of Incubation

After the fertilized egg leaves the mother, the development of the blastodermal cells is influenced by environmental factors, and embryos will sometimes stop developing when they are at 20.5°C (Edwards, 1902). Embryonic development is also affected when embryonic eggs are stored at constant temperature conditions (Özlü et al., 2018). Pre-incubation, temporary heating of the eggs before hatching, a method thought to mimic the natural conditions provided by birds for eggs before they start to hatch (Meijer and Siemers, 1993), allows embryos to form hypocotyls before hatching, reduces the negative effects of storage time and improves hatchability (Fasenko et al., 2001a; Fasenko et al., 2001b). It was found that embryonated eggs heated four times at 37.5°C at four-day intervals during 21 days of storage increased the body weight of broiler chicks before and after hatching compared to single heating for 6 h or 12 h during storage (Dymond et al., 2013). Embryonated eggs heated for 4 h at 30°C under 12 days of storage increased the body weight of broiler chicks at 21 D, and this positive effect on body weight could be sustained up to market age (Damaziak et al., 2018).

#### 2.5.3 Egg Turning

In artificial incubation of domestic poultry eggs, the angle of egg turning is also crucial to achieve high hatching performance (Deeming, 2009). Turning prevents inappropriate adhesion of the embryo to the inner shell membrane or of the allantoic sac to the yolk sac early in embryonic development (Cutchin et al., 2009). Stimulating vasodilation of the yolk sac membrane improves blood circulation and accelerates nutrient uptake into the yolk, thus promoting embryonic development (Deeming, 1989a,b). During egg incubation, a typical flip angle set at 45 degrees per hour can better promote embryonic development (Wilson, 1991). During gosling egg incubation, adjusting the rotation angle of the incubator to 70 degrees upregulated *GHRH*, *GH*, and *IGF-1* mRNA expression in E29 gosling embryos and also promoted *Pax7*, *MyoD*, and *MYF5* mRNA expression in the leg muscles, increasing embryo and leg muscle weight of E29 and increasing the cross-sectional area of leg muscle myofibrils and increasing at hatching weight. Therefore, increasing the rotation angle of the incubator was able to increase the number of myogenic cells and satellite cell number activity in muscles in the late incubation period, beneficial to embryonic muscle development.

#### 2.5.4 Electromagnetic Fields

The increasing number of various types of radiofrequency electromagnetic fields used for wireless communication in the living environment requires a critical assessment of their potential health effects, and the chicken embryo has traditionally been considered an ideal model for studying the effects of environmental factors. Electromagnetic stimulation providing various parameters during incubation has no effect on chicken morphology. There is no effect on tissue or gene expression (Woelders et al., 2017). However, another study showed that microwave frequency electromagnetic fields produced stress on embryonic cells and increased Hsp70 protein levels without causing a significant temperature increase (Shallom et al., 2002). However, it is not clear whether electromagnetic fields have an effect on embryonic muscle fiber development and posthatch meat quality.

## 3 Future Directions and Perspectives

Despite extensive research on myofiber development in avian embryos, it is unclear how environmental factors during incubation regulate myofiber development. Myofiber development is closely related to the myoblast dynamics including proliferation, differentiation and apoptosis. The role of incubation environment on the myoblast dynamics in developing embryos and early chicklings is worthy to be explored (Rozenboim et al., 2003; Zhang et al., 2012). The persisting or lasting effects of hatching environmental factors on muscle fibers transformation and meat quality on market age chickens also warrants further research. Furthermore, the synergistic or combinative effects of different incubation environmental factors on muscle development and meat quality are needed to investigated, which will lay the foundation for embryonic environmental regulation of muscle development and meat quality from theory to production.

It is worth noting that the modulation of incubation temperature has good industrial prospects on the alleviation of muscle disease in birds, as evidenced by the reduction of adverse effects of heat stress on meat quality. Muscle is high quality protein source, that is, popular with consumers worldwide, including some tropical regions. However, broilers kept under high temperature conditions may suffer from heat stress and have delayed muscle growth and adverse meat quality (Song and King, 2015; Gonzalez and Jackson, 2020). Compared with the normal incubation temperature, increasing the incubation temperature (38.8–39.5°C) could make broilers to be heat tolerant after shelling, thereby reducing susceptibility and severity to common myopathy and subsequently, improving the meat quality (Janisch et al., 2015; Morita et al., 2016; Clark et al., 2017). It is of value that an appropriate increase in incubation temperature could be applied in the poultry industrial hatching process and used to mitigate the negativity associated with high temperatures during muscle growth and development.

The cause of woody breast, white striping and spaghetti meat may be related to imbalance between the fast breast muscle growth rate and lagging cardiovascular systems development (Kawasaki et al., 2018; Zaboli et al., 2019). As hypertrophic breast muscle led to a reduced capillary density, this vascular hypoplasia may result in an impaired oxygen supply and metabolic waste product displacement from breast myofibers (Sihvo et al., 2018). This condition may exacerbate the occurrence of venous inflammation and become a major cause of muscle abnormalities (Papah et al., 2017). Although embryos in a hypoxic environment have faster cardiovascular systems development and better nutrient delivery to the pectoral muscle, whether moderate hypoxia can improve woody meat, white striping and spaghetti of pectoral muscle by promoting vascular development to the chest is unclear and needs to be further investigated.

## 4 Conclusion

Storage of fertile egg before incubation and incubation conditions affect embryonic muscle development and meat quality in poultry. During storage, embryos development can be stalled, and prolonged storage can lead to changes in the physical and chemical properties of embryonic components, affecting embryonic activity and muscle fiber development, which is detrimental to muscle growth and final meat quality, but short phase of incubation during egg storage can improve the ability of embryos to resume development and reduce the harmful effects of storage time on embryo development (Dymond et al., 2013). During incubation, changes in light, temperature and other condition parameters may alter myofiber development by regulation of hormones and growth factors as well myogenic regulatory factors, which may contribute to the improvement of final meat quality. Although, the precise actions of incubation environmental factors on myofiber development during incubation is still unclear, the muscle fiber metabolism and transformation is warranted for further research.

Nevertheless, the manipulation of environmental factors during the incubation phase is convenient and low-cost, which may avoid the stress of various regulatory approaches on birds after emergence, and is in line with the requirements and development of animal welfare. We have reasons to believe that the regulation of incubation conditions may benefit the poultry industry by stimulation of muscle fiber development and improvement of meat quality. Therefore, as more accurate and reliable studies become available, the role of embryonic environmental changes in regulating muscle fiber development and meat quality will play an increasingly important role in the meat poultry production industry.

## Abbreviations

E, embryonic day; D, day of age; T3, thiothyrotropine; T4, thyroxine; GHRH, growth hormone-releasing hormone; GH, growth hormone; IGF-1, insulin-like growth factor 1; GHR, growth hormone receptor; MyoD, myogenic differentiation antigen; Myf5, myogenic factor-5; MyoG, myogenin; Pax7, paired box 7; MRF4, myogenic regulatory factor-4.
